# Host gene expression changes in cattle infected with Alcelaphine herpesvirus 1

**DOI:** 10.1016/j.virusres.2012.08.011

**Published:** 2012-10

**Authors:** George C. Russell, Julio Benavides, Dawn M. Grant, Helen Todd, Jackie Thomson, Vipul Puri, Mintu Nath, David M. Haig

**Affiliations:** a Moredun Research Institute, Pentlands Science Park, Midlothian EH26 0PZ, UK; b Biomathematics and Statistics Scotland, JCMB, The King's Buildings, Edinburgh EH9 3JZ, UK; c School of Veterinary Medicine and Science, Nottingham University, Sutton Bonington, Leicestershire LE12 5RD, UK

**Keywords:** Malignant catarrhal fever, Microarray, Wildebeest, Cattle, Disease, Pathogenesis

## Abstract

Malignant catarrhal fever is a lymphoproliferative disease of cattle and other ungulates caused by infection with gamma-herpesviruses of the genus *Macavirus*. These viruses do not establish a productive infection but instead replicate in a cell-associated fashion in T lymphocytes, leading to systemic immune dysregulation and a generally fatal outcome. Despite significant progress in understanding the pathology of this disease, its pathogenesis remains unclear.

To identify genes and pathways affected in clinical MCF, sixteen bovine GeneCHIP microarrays were used to assay RNA from kidney and lymph node of four MCF-affected and four control *Bos taurus* steers. This is the first expression study of AlHV-1-MCF in the bovine host. Over 250 genes showed significant changes in gene expression in either lymph node or kidney, while expression of 35 genes was altered in both tissues. Pathway and annotation analysis of the microarray data showed that immune response and inflammatory genes were up-regulated in the kidney while proliferation-associated transcripts were additionally increased in the lymph node. The genes that showed the largest expression rises in both diseased tissues included cytotoxic enzymes and pro-inflammatory chemokines.

These data are consistent with disease-induced stimulation of inflammatory responses involving interferon-γ, including cytotoxic T cell recruitment and activation in peripheral tissues containing virus-infected cells. However it remains unclear whether the tissue damage in MCF lesions is due entirely to the activity of infected cells or whether uninfected T cells, recruited and activated at lesion sites through the action of infected cells, contribute to the pathogenesis of MCF.

## Highlights

► Microarray expression study of AlHV-1 infection in the natural (bovine) host. ► 35 genes had significantly altered expression in MCF-affected lymph node and kidney. ► Cytotoxic effector genes were up-regulated in both tissues. ► T cell chemokines and inflammatory cytokines were up-regulated in both tissues. ► Results consistent with chronic inflammatory pathology involving IFNγ.

## 1 Introduction

Malignant catarrhal fever (MCF) is a dramatic, fatal disease of cattle and other ungulates, including deer, bison and pigs, caused by ruminant gamma-herpesviruses of the genus *Macavirus* including *Alcelaphine herpesvirus 1* (AlHV-1) and *Ovine herpesvirus-2* (OvHV-2) (Loken et al., 1998; Reid et al., 1984; Schultheiss et al., 2000). The disease is characterised by fever and inappetence, frequently with ocular and nasal discharge, and is usually fatal. Death can occur within a few days or up to several weeks after the onset of clinical signs. There is no known treatment or licensed vaccine for MCF but a vaccine strategy to protect animals from MCF caused by AlHV-1 has been described recently (Haig et al., 2008; Russell et al., 2012).

AlHV-1 causes MCF mainly in East Africa, following the range of the wildebeest (*Connochaetes taurinus*), the natural host species, which propagates the virus without apparent clinical signs (Russell et al., 2009). Wildebeest-associated MCF (WA-MCF) is a significant issue for pastoralists in Eastern and Southern Africa, where cattle are moved to avoid contact with grazing or calving wildebeest (Bedelian et al., 2007; Cleaveland et al., 2001). WA-MCF also has been a problem in zoological collections that contain wildebeest (Meteyer et al., 1989; Whitaker et al., 2007). OvHV-2 naturally infects sheep and causes sheep-associated MCF (SA-MCF), which is found worldwide wherever sheep and cattle (or other MCF-susceptible species) are kept together. SA-MCF is a particular problem in North America where bison are highly susceptible to infection (Berezowski et al., 2005).

An interesting feature of MCF is that the natural reservoir species for the viruses causing MCF (*e.g.* wildebeest and sheep) do not exhibit any clinical signs of infection, whereas the disease is dramatic and usually fatal in MCF-susceptible species, some of which are phylogenetically closely related to the reservoir hosts. There are excellent animal models of MCF. In particular, rabbits can be infected with AlHV-1 or OvHV-2 and develop MCF that is very similar to that seen in species naturally susceptible to MCF (Anderson et al., 2007; Buxton et al., 1984). This model of MCF is now widely used in the analysis of MCF pathogenesis, including analysis of recombinant AlHV-1 (Anderson et al., 2008; Boudry et al., 2007; Dewals et al., 2006, 2011, 2008; Li et al., 2011; Meier-Trummer et al., 2009b). Analysis of the pathology of MCF induced by AlHV-1 and OvHV-2 suggests an immune-mediated aetiology, with infiltration/proliferation of lymphocytes in multiple tissues (Anderson et al., 2007). Recent studies have suggested that the proliferating cells are T cells and that many of these may be infected (Dewals et al., 2008; Nelson et al., 2010). Infected large granular lymphocyte (LGL) cell lines that can be propagated in culture from MCF-affected animals have a non-MHC-restricted cytotoxic T cell phenotype similar to lymphokine-activated killer cells and it has been suggested that these cells cause the dramatic pathology seen in MCF (Anderson et al., 2008; Schock et al., 1998; Swa et al., 2001). The exact aetiology of MCF pathogenesis has yet to be elucidated.

The involvement of virus gene expression in disease progression has been studied mainly in the rabbit model, where different conclusions have been drawn in different studies. Thus, studies of OvHV-2 infection by intra-nasal or intravenous inoculation have demonstrated lytic cycle-associated gene or protein expression in a range of tissues (Li et al., 2011; Meier-Trummer et al., 2009b), while analysis of lymphoid tissues from rabbits infected intravenously with AlHV-1 has shown a latency-associated gene expression pattern (Dewals et al., 2011, 2008). The inability of either virus to produce infectious virus particles in MCF-susceptible species also suggests that the full programme of lytic virus gene expression is not completed in these hosts. Thus the virus may be limited to some form of latency or to an aberrant pattern of gene expression that is abortive for virus propagation. Analysis of LGL from cattle and rabbits infected with OvHV-2 showed that these cells contained circular (latent) genomes and did not express known lytic cycle transcripts, although some viral gene expression was detected (Thonur et al., 2006). The conflicting evidence of recent studies may also suggest that virus gene expression patterns can vary depending on host species and cell type infected.

Virus-specific and bovine genome microarrays have been used recently to identify virus and host genes whose expression was altered in lymph node tissue of two cattle naturally infected with OvHV-2 (Meier-Trummer et al., 2009a). This study found that virus infected cells in MCF-affected cattle had a latent pattern of gene expression; that cytotoxicity and cell-cycle associated genes were up-regulated; and notably that IL-2 expression was reduced by about 7-fold on average. Other work has suggested the involvement of IL-15 in the proliferation and cytotoxic activity of infected cells in AlHV-1 induced MCF (Anderson et al., 2008) but the crucial factors in MCF pathogenesis have yet to be confirmed *in vivo*.

Recent work in this laboratory has led to the development of an intranasal route for infection of cattle with AlHV-1 that produces reliable induction of MCF. This has been used in the development of a protective vaccine strategy for MCF in cattle (Haig et al., 2008; Russell et al., 2012) and has provided tissues for detailed analysis of MCF pathology (J. Benavides, H. Todd, G.C. Russell, D.M. Haig, unpublished data). In this paper we describe the use of lymphoid and non-lymphoid tissues from this natural infection of MCF to characterise patterns of gene expression involved in disease pathogenesis. Host gene expression changes due to MCF were identified using RNA extracted from kidney and prefemoral lymph node tissue samples from infected and uninfected cattle. The kidney was used as it represents the cellular pathological changes seen in non-lymphoid tissues in MCF-affected animals. Uninfected kidney contains few lymphoid cells, so the infected tissue may reveal MCF-specific effects that are not distinguished in lymphoid tissue, where the background of lymphoid cells not involved directly in MCF may confound transcriptional analysis. Microarray analysis was used to provide data on approximately 23,000 genes and bioinformatic analysis was used to identify significantly affected genes (up- or down-regulated compared to control samples), functional classes and pathways, shedding light on potential mechanisms of pathogenesis.

This is the first report of gene transcription analysis in a natural disease-susceptible species (cattle) infected with AlHV-1. This should allow any differences and similarities to AlHV-1-infected rabbits or OvHV-2-infected cattle to be revealed.

## 2 Materials and methods

### 2.1 Animals and tissues

The animal experiments were carried out with the approval of the Moredun Research Institute Experiments and Ethical Review Committee and complied fully with the Home Office of Great Britain and Northern Ireland “Animals (Scientific Procedures) Act 1986”.

Disease-free and OvHV-2 seronegative male Friesian-Holstein cross calves between 3 and 5 months of age were used. One group of seven cattle was infected intranasally with 10^4.55^ TCID50/ml (50% tissue culture infectious dose) of pathogenic AlHV-1 as described previously (Haig et al., 2008; Russell et al., 2012) while a control group of four animals was left uninfected. The cattle were monitored daily and animals exhibiting a rise in rectal temperature to over 40 °C for at least 2 days with other clinical signs consistent with MCF (inappetence and ocular signs or palpable lymphadenopathy) were euthanised with an overdose of intravenous sodium pentobarbitone. Diagnosis of MCF in these animals was confirmed by gross and histopathological examination of tissue samples and by diagnostic PCR detection (Traul et al., 2005) of AlHV-1 DNA in blood samples taken at post-mortem. Samples of prefemoral lymph node (LN) and renal cortex (Kid) were collected asceptically from four infected and four control animals and were stored at −20 °C in RNAlater (Life Technologies) as cubes smaller than 5 mm.

### 2.2 RNA extraction and microarrays

Tissue samples of about 100 mg from control uninfected and MCF affected cattle were used for RNA preparation using Trizol reagent and a silica-based RNA purification system (Qiagen) according to the protocol of ARK-genomics (www.ark-genomics.org). RNA samples from both tissues of 4 MCF-affected and 4 control animals that passed the RNA quality criteria (*A*_260/280_ ratio = 2.0 ± 0.05; RNA integrity number (RIN) > 7.0 as assessed using the Eukaryotic RNA 6000 LabChip kit on the Agilent 2100 Bioanalyser) were submitted to ARK-genomics for microarray analysis using Affymetrix GeneChip^®^ Bovine Genome Arrays, representing about 23,000 transcripts.

### 2.3 Data processing

The data were pre-processed using the MAS 5.0 background adjustment method as outlined in the Affymetrix Statistical Algorithms Description Document (http://media.affymetrix.com/support/technical/whitepapers/sadd_whitepaper.pdf). To make measurements from different arrays comparable, data were normalised by the quantile normalisation method (Bolstad et al., 2003). Quality assessments of probe-level Affymetrix chip data as well as normalised data were carried out using statistical and graphical methods including MA plots and smoothed histogram plots. The MA-plots showed normalisation was effective; while smoothed histograms indicated there was no systematic bias in the data. The smallest statistically significant difference in mean expression level between affected and control microarrays was about 1.3-fold, demonstrating the reliability of the microarray data presented here. The raw and normalised data from this experiment have been submitted to the ArrayExpress database and have been assigned the accession number E-MEXP-3497.

### 2.4 Gene expression analysis

Gene expression measurements were managed following the MIAME standard (Brazma et al., 2001). A general linear model was fitted to the normalised expression data for differential expression analysis. The coefficients of the fitted models corresponded to two types of RNA sources (tissue type: kidney and lymph node; treatment type: MCF-affected and control). The test statistic for each gene was the moderated *t*-statistic, where the standard error used to calculate the *t*-statistic was estimated using the posterior obtained from an empirical Bayesian model (Smyth, 2004). The overall significance of each test was adjusted using the false discovery rate (FDR) approach of Benjamini and Hochberg (1995). All analyses were carried out using the Bioconductor suite of programs under the R 2.10.1 environment (www.r-project.org). Probesets with statistically significant differences in mean expression (FDR-adjusted *p*, *p*_*f*_ < 0.01 and mean fold-change (FC) > 2.0) in MCF-affected *versus* control tissues were identified as being suitable for further analysis. These probesets were further analysed using the Ingenuity Pathways Analysis (IPA) system (www.ingenuity.com) and the DAVID online software system (Huang et al., 2008).

### 2.5 Real-time quantitative PCR of selected genes

Three host genes with significant up-regulation of expression in MCF-affected animals and two genes with no evidence of expression change were selected for real-time PCR confirmation of microarray data. RNA extracted from MCF-affected and control tissues for microarray analysis was also used for these assays to facilitate comparison. The real-time PCR primer and probe sets for each gene were designed using Primer Express software (Applied Biosystems, Warrington, UK) (Table 1). Where possible, primer-probe sets were designed to span exons within the respective genes to reduce detection of genomic DNA contamination of RNA. All primers and probes were designed within a narrow annealing temperature range to facilitate the optimisation of the PCR and the simultaneous analysis of multiple genes. Primer and probe optimisation was done according to the instrument manufacturer's protocols, using a chequerboard of forward and reverse primer concentrations and titration of probe concentration. Concentrations of primers (900 nM) and probes (250 nM) that gave the lowest threshold cycle (Ct) values and highest fluorescence yield (ΔRn) across a range of concentrations of pooled RNA template were used in all subsequent assays. The real-time quantitative (q)RT-PCR was carried out using the Superscript III platinum One-Step qRT-PCR kit (Life Technologies). Reactions of 20 μl each contained: 10 μl of 2× Reaction Mix; 2.0 μl of each 9 μM primer; 1.0 μl of 5 μM probe; 4.0 μl PCR grade DNase-free water and 1.0 μl (100 ng) total RNA. Prior to analysis, RNA samples were treated with amplification-grade DNAse I (Life Technologies) to reduce background amplification due to genomic DNA contamination. All reactions were carried out on a Roche LightCycler (Roche Applied Science) with the following cycling parameters: 50 °C for 15 min and 95 °C for 2 min followed by 40 cycles of 95 °C for 15 s and 60 °C for 30 s. Data were analysed using LightCycler 480 software (version LCS480 1.5.0.39).

Additionally, parallel control amplifications were performed on each RNA sample in the absence of a reverse transcription step to confirm that genomic DNA contamination did not contribute to the amplification seen.

Bovine ribosomal protein S9 (RPS9) was chosen as the reference gene for these studies because its mean expression was not statistically significantly altered in either tissue in response to MCF in the microarray study. The use of this gene as a gene expression standard has also been validated in multiple cattle tissues under different metabolic conditions (Bionaz and Loor, 2007; Janovick-Guretzky et al., 2007) and in neoplastic *versus* normal tissues (Blanquicett et al., 2002).

Analysis of gene expression was performed by relative quantification, normalised to the reference gene RPS9, using the ΔΔCt method as described (Schmittgen and Livak, 2008). Briefly, mean Ct values were derived from replicate assays for each gene and sample (tissues: lymph node and kidney; conditions: MCF and control). Next, for each gene, tissue and condition the mean Ct for the RPS9 reference gene was subtracted from the mean Ct for each gene assayed, giving a ΔCt value for that gene/tissue/condition. The ΔCt values for the control group were then subtracted from the respective MCF ΔCt values for each gene and tissue, giving a ΔΔCt value that represented the fold-change between MCF and control animals. Finally, the actual fold-change values between MCF and control samples for each gene and tissue were calculated as 2^−ΔΔCt^.

## 3 Results

### 3.1 Induction of MCF

All animals in the group inoculated intranasally with pathogenic AlHV-1 showed clinical signs of MCF at 27–30 days post-infection. The animals were euthanised on the second or third day of pyrexia, with additional clinical signs consistent with MCF (conjunctivitis or lymphadenopathy). The diagnosis of clinical MCF was confirmed by gross and histopathological findings and by the detection of AlHV-1 virus DNA in blood and tissue samples. Histologically, the kidney from the infected animals showed multifocal interstitial nephritis characterised by non-purulent vasculitis, mainly affecting the arcuate vessels although lesions were also visible in other minor veins and arteries. The lymph node from these animals showed a diffuse follicular lymphoid hyperplasia. Samples from the non-infected animals showed no gross or histological lesions. No clinical signs were seen in the control animals, which were subject to post-mortem examination separately from the MCF-affected animals.

### 3.2 Identification of differentially expressed genes in MCF-affected tissues

Analysis of gene expression in two tissues from four animals each in control or MCF-affected groups was done using a single-dye approach on 16 Affymetrix GeneChip Bovine Genome Arrays (www.affymetrix.com/analysis/index.affx). RNA for microarray analysis was extracted from tissue samples using a standard protocol and was stored in aliquots at −80 °C. Assessment of RNA integrity by capillary electrophoresis ensured that all RNA used for this analysis was of a similar high quality. The RNA samples submitted for microarray analysis had concentrations between 1.1 and 2.3 mg/ml, *A*_260/280_ ratios of 2.00–2.02 and RIN values of 7.4–8.9. Analysis of hybridisation intensity data after background adjustment and normalisation showed that there was little systematic bias in the observed data.

The array data for each set of four samples was combined and used to identify probesets with statistically significantly different mean expression levels between control and MCF-affected samples within each tissue (*p*-value adjusted for false discovery rate (FDR), *p*_*f*_ < 0.01). Over 1350 probesets were statistically significantly altered in lymph node while more than 760 were found to be statistically significantly altered in kidney. Of these, 197 probesets in lymph node and 291 probesets in the kidney had average fold-change differences of more than 2. These probesets corresponded to 269 currently annotated genes of which 69 genes were up-regulated by at least 2-fold on average in lymph node compared with 161 genes in the kidney; while 52 genes showed at least 2-fold average down-regulation in the lymph node compared to only 22 genes in the kidney (supplementary Table S1). Interestingly, only 35 genes showed altered expression in both tissues (Table 1), of which 32 genes were up-regulated. This observation suggests that MCF may induce tissue-specific changes in gene expression.

The observation that most of the genes with altered regulation in both tissues increased in expression during MCF suggests the induction of a core gene set that may be illustrative of the major changes in gene expression that occur in MCF (Table 2). Of this group of genes, those with the greatest degree of change included: granulysin (GNLY); granzyme H (GZMH); chemokine (C–X–C motif) ligand 11 (CXCL11); ubiquitin D (UBD); chemokine (C–C motif) ligands 3, 4 and 5 (CCL3, CCL4 and CCL5); the CD8 alpha chain (CD8a); placenta-specific 8 (PLAC8); and indoleamine 2,3-dioxygenase 1 (IDO1). All of these genes have known functions relating to regulation of immune responses and may therefore have roles in the immune dysregulation that characterised MCF.

In contrast, only 3 genes were found to show more than 2-fold reduced expression in both tissues of MCF-affected animals. These genes: cold inducible RNA binding protein (CIRBP); stromal antigen 3 (STAG3); and RNA binding motif protein 3 (RBM3) have known functions related to chromosome pairing (STAG3) and the response to metabolic insults (*e.g.* cold). These differences may be a consequence of MCF, driven either by pathogenic processes or the anti-viral response, but their potential role in disease is unclear. In order to integrate the contributions of multiple genes to MCF pathogenesis and potentially identify biological processes or pathways that may be co-regulated in the diseased tissues, global analysis of up- and down-regulated genes was performed using two systems with distinct approaches.

### 3.3 Quantitative PCR analysis of gene expression changes

Real-time quantitative (q)PCR was used as an independent assay to verify that the microarray analysis had correctly identified genes with significant expression changes between control and MCF-affected tissues. Five genes were selected for analysis on the basis of microarray data and qPCR primer-probe sets were designed for each gene (Table 1). Two genes were selected with the highest expression change in both tissues (GNLY, GZMH; Table 2); two genes were selected that had unchanged expression (glucose-6-phosphate isomerise (GPI), RPS9); and one gene was selected that had a high level expression change in one tissue (granzyme B (GZMB); supplementary Table S1). No genes with reduced expression were selected for analysis by this method as the expression change in these genes (2–3-fold reduction) was considered to be too low to reliably distinguish by real-time PCR (Table 2).

For this analysis RPS9 was selected as the reference gene because it had previously been validated for quantitative analysis in bovine tissues (Bionaz and Loor, 2007) and it exhibited little variation in the microarray analysis (mean fold-change in MCF-affected tissue was 1.03 in kidney and 1.07 in lymph node with *p*_*f*_ values of 0.84 and 0.54 respectively). Quantitative PCR analysis of RPS9 expression in the tissues examined (kidney and lymph node; infected and control) revealed low levels of variation. Ct values for assays of equivalent amounts of total RNA had a standard deviation of less than 1 cycle, supporting the use of RPS9 as a reference gene in this analysis. RNA samples were assayed in duplicate or triplicate and mean Ct values were used to calculate fold-change differences in expression of the selected genes between MCF and control tissues using the ΔΔCt method (Schmittgen and Livak, 2008).

**Fig. 1:**
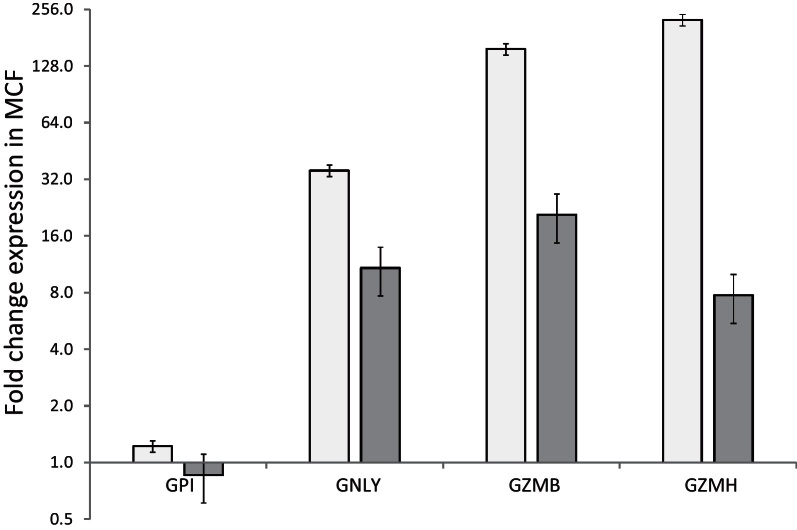
Gene expression fold-change values derived from qPCR data. The changes in expression of four genes: glucose-6-phosphate isomerase (GPI); granulysin (GNLY); granzyme B (GZMB); and granzyme H (GZMH) in MCF-affected tissues were normalised to ribosomal protein S9 (RPS9) and plotted with respect to equivalent tissues from control animals. Results of kidney transcript assays are in the light columns while results of lymph node assays are in the dark columns.

These results (Fig. 1) verified the up-regulation of GNLY and GZMH, which showed greater than 5-fold increases in mean expression in both tissues, and suggested that expression of GZMB was also increased in both tissues. From the microarray data for the gene GZMB, a statistically significant change in the mean expression level was found in the lymph node (FDR-adjusted probability, *p*_*f*_ = 0.0001). In addition, the *p*_*f*_ value of 0.028 provided weak evidence for an altered mean expression level of GZMB in kidney (fold change of 2.5). Although this did not attain formal statistical significance as defined by the chosen threshold of *p*_*f*_ < 0.01, it provided some support for the real-time PCR results suggesting that GZMB was up-regulated in both tissues.

The fold-change estimated by real-time PCR for GNLY, GZMB and GZMH was greater than that suggested by the microarray data, especially for the kidney RNA samples. This is in accord with comparative analyses, which observed that fold change results determined by qRT-PCR were greater than those determined by microarray analysis (Dallas et al., 2005; Yuen et al., 2002). Expression of the housekeeping gene GPI, in contrast to the cytotoxic genes GNLY, GZMH and GZMB, was not affected by MCF, varying by less than 2-fold in either tissue (Fig. 1).

These data support the view that the microarray analysis was able to correctly identify significant changes in gene expression due to MCF.

### 3.4 Analysis of enriched gene ontology terms in differentially expressed genes

The on-line database system DAVID (Huang et al., 2008, 2009) was used to identify gene ontology terms, functional classes and pathways that were enriched among the differentially expressed genes in each tissue. This analysis is summarised in Table 3. DAVID analysis uses only gene identifiers, so separate datasets for up- and down-regulated genes were analysed for each tissue, comprising those genes with *p*_*f*_ < 0.01 and mean fold-change (FC) greater than 2. Highly enriched annotation terms among up-regulated genes in the kidney included: antigen presentation; inflammatory responses; immune regulation and activation. These processes were also reflected in up-regulated pathways such as antigen presentation, graft rejection and graft-*versus*-host disease, all suggestive of increased immune activity in this tissue (Table 3). This contrasts with the enriched terms relating to up-regulated genes in the lymph node (cell division and mitosis in addition to chemokines and autoimmunity) suggesting active immune responses and proliferation. Annotations enriched among down-regulated genes in kidney were fewer in number and of lower significance, reflecting changes in biosynthetic processes, while the annotations corresponding to lymph node down-regulated genes included cell adhesion, control of proliferation by follistatins as well as some biosynthetic processes (Table 3).

It is clear from the observed pathology of MCF that non-lymphoid organs experience a significant influx of lymphoid cells (including virus-infected cells) which contribute to lesion development. Thus the annotation enrichment for kidney is likely to reflect the transcriptional activity of activated lymphocytes that are responsible for the observed pathology of MCF. Lymph node hyperplasia is a known element of MCF pathology (Anderson et al., 2007; Buxton et al., 1984; Jacoby et al., 1988) and this is reflected in the annotation enriched in this tissue. Thus the tissue-specific differences in enriched annotation in response to MCF may reflect both disease-specific changes in gene expression and changing cell populations in the different tissues.

### 3.5 Pathway analysis of differentially expressed genes

Differentially regulated pathways were also investigated using Ingenuity Pathway Analysis. Briefly, *p*_*f*_ and FC values associated with the entire dataset of lymph node and kidney were uploaded to the IPA software for global analysis. Use of the entire dataset allowed those genes with small but significant changes in mean expression to contribute to the identification of significantly altered gene groups and pathways. It also allows the background of genes that did not change in expression to contribute to the evaluation of potentially significant pathways. The main findings of this analysis are summarised in Table 4.

This analysis also highlights differences between kidney and lymph node gene expression patterns, especially in the networks, disorders and pathways that were identified as having significant scores or *p*-values. The most significant networks/disorders identified included those associated with cancer and cell proliferation in the lymph node; and antigen presentation and inflammatory responses in the kidney (Table 4). Indeed, across a range of analyses performed with the IPA database (Table 4), there appears to be a clear difference in the balance of transcriptional activity induced by MCF. The lymph node sees changes in functions associated with cell proliferation and growth regulation, while changes in the kidney relate largely to increased immunological activity as well as proliferation. Significant up-regulation of pathways relating to immune function is seen only in the kidney (antigen presentation; CD28 signalling; iCOS–iCOSL signalling; Natural Killer cell signalling), identifying activated T lymphocytes and NK cells as likely effectors of tissue damage in MCF (Table 4). Thus the observed transcriptional changes in the kidney appear to reflect an increase in the expression of activated lymphocyte phenotypes, while those in the lymph node indicate strong proliferative activity, potentially among resident lymphocytes.

## 4 Discussion

In this study, gene expression changes induced by AlHV-1 infection of a natural MCF-susceptible host (cattle) were analysed using microarrays carrying probes for approximately 23,000 bovine transcripts. Individual microarrays were used to interrogate RNA samples from lymph node and kidney of multiple cattle. Kidney was chosen as a non-lymphoid tissue in which MCF induces substantial lymphoid infiltration, forming visible lesions (Russell et al., 2009), while the inclusion of lymph node tissue in the analysis was intended to allow local changes in immune effector functions to be distinguished from more general mitogenic changes that are a consequence of lymphoproliferation. The use of both lymphoid and non-lymphoid tissues, therefore, may also aid the interpretation of the data compared to the use of either individual tissue. The observation that more genes were found with significantly altered expression due to MCF in the kidney compared to lymph node supports the view that the kidney is an appropriate tissue in which to define the effects of MCF in cattle.

Initial analysis of the microarray results identified 35 genes whose mean expression was significantly increased in both MCF-affected tissues (Table 2A). These genes encode a range of proteins, including immunological effector molecules and chemokines. Closer examination of these highly expressed genes may provide clues to MCF pathogenesis.

One group of up-regulated genes encoded cytotoxic effector molecules. These included granulysin, granzyme H and perforin (GNLY, GZMH and PRF1; Table 2), while granzyme B (GZMB) was found to be up-regulated in kidney by microarray analysis and in both tissues by qRT-PCR (supplementary Table S1, Fig. 1). This group included the most highly up-regulated genes in both tissues, underlining their significance in the generation of MCF pathology.

Chemokine ligands formed another group of up-regulated genes. CCL3, CCL4, CCL5 and CXCL11 were up-regulated in both tissues while CCL8, CCL19 and CXCL10 were up-regulated in kidney. These are all pro-inflammatory chemokines that function by recruiting and activating T cells and other lymphocytes. In particular, CXCL10 and CXCL11 bind the receptor CXCR3 on activated T cells to recruit them to peripheral sites. These chemokines and their receptor are associated with T cell trafficking in a range of inflammatory conditions and autoimmune diseases (Groom and Luster, 2011). They may therefore play a role in the T cell infiltration that appears to be a central feature of MCF pathogenesis. A CXCR3 antagonist compound has been shown to inhibit chemotaxis and attenuate disease in a range of autoimmune models (Jenh et al., 2012). MCF can be viewed as a disease exhibiting similarities to autoimmune pathogenesis that makes the CXCR3 receptor and its ligands interesting targets for future research and potential therapies.

Several of the genes in Table 2A are inducible by IFNγ (*e.g.* CCL3, CCL4, CCL5, CXCL11, IDO, UBD), suggesting that this may be a common factor underlying the pathogenesis of MCF. IFNγ itself was up-regulated by over 3-fold in the tissues analysed here (Table 2), supporting the view that it may be responsible for many of the expression changes observed. Indeed, aberrant expression of IFNγ has been associated with inflammatory and autoimmune diseases (Schoenborn and Wilson, 2007).

A previous microarray analysis of gene expression in MCF-affected lymph node from OvHV-2 infected cattle (Meier-Trummer et al., 2009a) showed that IL-2 expression was reduced in MCF-affected lymph node and suggested this might be a key to understanding MCF pathogenesis. This observation was confirmed by qPCR studies in AlHV-1 infected rabbits (Dewals and Vanderplasschen, 2011), that also detected up-regulation of IFNγ, perforin and IL-10, suggesting that the pathology of MCF was similar in rabbits infected with either of the major MCF viruses. The data presented here in AlHV-1-infected cattle confirmed the increased expression of pro-inflammatory cytokines and cytotoxic effectors in MCF (Table 2), but provided no evidence for changes in expression of IL-2 or IL-10.

This could reflect a genuine difference in AlHV-1-infected cattle compared to OvHV-2-infected cattle, or MCF-affected rabbits. Alternatively, the expression differences observed may reflect the challenge system used in this work, since MCF induced by intranasal challenge has a longer incubation time than intravenous challenge (Haig et al., 2008) and the animals used here were euthanised at a relatively early clinical stage due to welfare requirements. Thus the load of infected cells in these tissues may have been lower than that in tissues used in previous work. Currently there is no simple, robust way of detecting and analysing virus-infected cells in the tissues of MCF-affected animals. Recent work using a recombinant MCF virus expressing luciferase illustrates one approach with the potential to address this issue in experimental systems (Dewals et al., 2011). In addition, we have recently identified a novel gene in AlHV-1 and detected its mRNA in MCF lesions by RT-PCR and *in situ* hybridisation (G.C. Russell et al., unpublished data). This may be a useful tool for the analysis of infected cell frequencies in the tissues.

Gene ontology and pathway analysis (Tables 3 and 4) suggested that immunological and inflammatory processes were increased in kidney compared with increased cell proliferation and trafficking in the lymph node. Non-reactive lymph node is likely to contain transcripts related to normal immunological processes, while kidney is not, so that transcription from lymphocytes recruited to the kidney as part of MCF pathogenesis is likely to make a greater impact on the pattern of gene expression than in the lymph node. However, the increased expression of cytotoxic effector genes and chemokine genes in the lymph node demonstrates that immunological dysregulation occurs in this tissue too, reflected in the observed lymph node pathology. This is in accord with reports that cytotoxic CD8 T lymphocytes infiltrate and proliferate in MCF lesions of bison and rabbits (Dewals and Vanderplasschen, 2011; Nelson et al., 2010) and that many of these cells are likely to be infected (Dewals et al., 2011; Dewals and Vanderplasschen, 2011).

The activities of the gene products over-expressed in both analysed tissues of the MCF-affected animals suggest that constitutive expression of inflammatory genes may contribute to the pathology of MCF (Table 2) with overexpression of IFNγ playing an important role. Thus, virus-infected lymphocytes, disseminated *via* the circulation to multiple organs, may act through expression of IFNγ-induced CXC and CC cytokines to recruit activated cytotoxic T cells to peripheral sites. We can speculate that the expression of high levels of cytotoxic effector molecules by virus-infected and/or uninfected cells in MCF-affected tissues generates a non-specific T cell cytotoxicity that leads to tissue damage.

Interestingly, infected large granular lymphocyte (LGL) lines that can be established from tissues of MCF-affected animals have non-MHC-restricted cytotoxic activity, are known to express pro-inflammatory cytokines and have constitutively active *Lck* and *Fyn* kinase pathways (Anderson et al., 2008; Schock et al., 1998; Swa et al., 2001). These cell lines have been suggested to represent the infected cells *in vivo*. The microarray data presented here adds further evidence for a connection between the phenotype of LGL lines and MCF pathogenesis. Strong evidence of cytotoxic activity, activated T cell signalling and the up-regulation of IFNγ expression are seen both in the LGLs and in MCF-affected cattle tissues (Schock et al., 1998; Swa et al., 2001).

In conclusion, this study of AlHV-1 induced MCF in cattle, a naturally susceptible host, confirms the results of previous studies of MCF in rabbits or OvHV-2-infected cattle with respect to a possible role for increased cytotoxicity, and for IFNγ-stimulated immune and inflammatory responses, but differs with respect to IL-2 depression. This requires further analysis, including assays to confirm that changes in mRNA expression are reflected at the level of protein expression and activity. The expression of up-regulated genes, such as GNLY, CXCL11, UBD or IFNγ, in MCF-virus-infected cells also warrants further study in order to clarify their role in pathogenesis—whether they act directly to initiate tissue damage *via* cytotoxic activity or indirectly by recruiting and activating cytotoxic T cells at lesion sites.

## Figures and Tables

**Table 1:** Primer and probe sequences used for quantitative PCR.

Gene symbol	Oligonucleotide sequence (5′–3′)	Accession number of cDNA
RPS9-F	GCCTCGACCAAGAGCTGAAG	DT860044
RPS9-R	GGGCAGCCTTTCGGATCT	
RPS9-P	FAM-TGATCGGCGAGTATGGGCTCCG-TAMRA	

G6PI-F	CATTGCCTCCAAGACCTTCAC	NM_001040471
G6PI-R	GGCCGACAGGAGAAACC	
G6PI-P	TET-ACCCAGGAGACCATCACGAACGCA-TAMRA	

GNLY-F	TGGGCTGTCCTGCTCATCA	NM_001075143
GNLY-R	TCAGGAGTCAGACCGGAAAAA	
GNLY-P	TET-CTCGGTGCTCCTGGTTGCCCC-TAMRA	

GRNZH-F	GCTGGGAGAGGAGATCATTGG	XM_002696688
GRNZH-R	CAGAAACTGAACGAAAGCCATGT	
GRNZH-P	TET-CCAAGCCCCACTCCCGCCC-TAMRA	

GRNZB-F	GTAGGTGCGGTGGGTTCCT	XM_002696646
GRNZB-R	TTGATTGAGCTTCCGTTGCA	
GRNZB-P	TET-TCGACAGGACTTCGTGCTGACAGCC-TAMRA	

**Table 2:** Known genes with at least 2-fold change between MCF-affected and control tissues.

Gene symbol^a^	Gene title^b^	K (FC)^c^	K (*p*_*f*_)^d^	LN (FC)	LN (*p*_*f*_)
**(A) Genes significantly up-regulated in both tissues of MCF-affected animals**					
GNLY	Granulysin (mean)^e^	8.9	0.0009	16.5	0.0002
GZMH	Granzyme H	10.2	<0.0001	15.1	<0.0001
CXCL11	Chemokine (C–X–C motif) ligand 11	14.3	0.0001	8.4	0.0009
UBD	Ubiquitin D (mean)	13.8	<0.0001	3.2	0.0080
CCL4	Chemokine (C–C motif) ligand 4	8.8	<0.0001	4.8	0.0002
CD8A	CD8a molecule	7.2	<0.0001	2.4	0.0007
CCL5	Chemokine (C–C motif) ligand 5	5.9	<0.0001	3.1	0.0002
PLAC8	Placenta-specific 8 (mean)	5.8	0.0001	5.6	0.0014
IDO1	Indoleamine 2,3-dioxygenase 1 (mean)	5.5	0.0001	4.9	0.0004
CCL3	Chemokine (C–C motif) ligand 3 (mean)	4.9	<0.0001	3.0	0.0008
OASL	2′–5′-oligoadenylate synthetase-like	2.2	0.0058	3.8	0.0003
HOPX	HOP homeobox	3.7	<0.0001	3.1	0.0004
FCGR3A	Low affinity Fc receptor IIIa, IgG (CD16a)	3.5	<0.0001	2.2	0.0011
WARS	Tryptophanyl-tRNA synthetase (mean)	3.5	<0.0001	2.3	0.0009
IFNG	Interferon, gamma	3.0	0.0024	3.3	0.0016
RRM2	Ribonucleotide reductase M2 polypeptide	3.2	0.0010	2.6	0.0048
PRF1	Perforin 1 (pore forming protein)	3.1	<0.0001	2.4	0.0006
CKS2	CDC28 protein kinase regulatory subunit 2	2.5	0.0015	3.1	0.0005
SH2D1A	SH2 domain protein 1A	3.0	<0.0001	2.7	0.0004
MTHFD2	Methylenetetrahydrofolate dehydrogenase 2	2.8	0.0003	2.3	0.0006
LMNB1	Lamin B1	2.3	0.0047	2.8	0.0010
CCR5	Chemokine (C–C motif) receptor 5	2.5	0.0001	2.5	0.0005
PPA1	Pyrophosphatase (inorganic) 1	2.5	0.0002	2.4	0.0006
NCAPG	Non-SMC condensin I complex, subunit G	2.5	0.0006	2.1	0.0029
ISG20	Interferon stimulated exonuclease gene 20 kDa	2.5	0.0012	2.4	0.0016
CDCA8	Cell division cycle associated 8	2.5	0.0005	2.2	0.0017
NUSAP1	Nucleolar and spindle associated protein 1	2.4	0.0021	2.1	0.0057
CD96	CD96 molecule	2.4	0.0003	2.4	0.0006
TOP2A	Topoisomerase (DNA) II alpha 170 kDa (mean)	2.4	0.0077	2.1	0.0090
UBE2C	Ubiquitin-conjugating enzyme E2 C	2.3	0.0004	2.2	0.0009
CCNB2	Cyclin B2	2.1	0.0051	2.1	0.0032
CRELD2	Cysteine-rich with EGF-like domains 2	2.0	0.0070	2.1	0.0048

Gene symbol	Gene name	K (1/FC)^f^	K (*p*_*f*_)	LN (1/FC)	LN (*p*_*f*_)
**(B) Genes significantly down-regulated in both tissues of MCF-affected animals**					
CIRBP	Cold inducible RNA binding protein	3.3	<0.0001	3.3	0.0002
STAG3	Stromal antigen 3 (mean)	2.5	0.0001	2.5	0.0004
RBM3	RNA binding motif protein 3 (mean)	2.0	0.0008	2.5	0.0002

a Gene identities for probesets from the Affymetrix GeneChip Bovine Genome Arrays were obtained from the NetAffx™ Analysis Center (www.affymetrix.com/analysis/index.affx) or were assigned following sequence-based database searching.

b Genes are ordered on the basis of their maximal expression change in either tissue.

c Fold-change in gene expression (FC) in MCF compared to control tissue, and *p*-values adjusted for false discovery rate (*p*_*f*_) are listed for kidney (K) and lymph node (LN) samples.

d False discovery rate adjusted *p*-values (*p*_*f*_) are expressed with 4-decimal precision.

e Where multiple probesets representing the same gene were significantly altered in expression, the FC value given is the mean, indicated in the gene name column, while the *p*_*f*_ value shown is the highest of the probesets analysed.

f Degree of down-regulation is expressed as the inverse of the fold-change (1/FC).

**Table 3:** DAVID software analysis.

Annotation type^a^	Kidney	Score b	*p*-Value c	Lymph node	Score	*p*-Value
Up-regulated annotation clusters	Antigen processing and presentation	6.0		Cell division	6.2	
	Inflammatory response	5.8		Mitosis, cell cycle	2.8	
	Regulation of immune response	5.3		Chemokines and receptors	2.4	
	Regulation of lymphocyte mediated immunity	3.6		Autoimmune reaction	2.4	
	Regulation of lymphocyte activation	3.5		Meiosis	2.0	

Up-regulated KEGG pathways	Antigen processing and presentation		<0.0001	Meiosis		0.0001
	Allograft rejection		<0.0001	Graft-*versus*-host disease		0.0013
	Graft-*versus*-host disease		<0.0001	Cell cycle		0.0018
	Viral myocarditis		<0.0001	Allograft rejection		0.0018
	Cell adhesion molecules		<0.0001	Cytokine-receptor interaction		0.0020

Down-regulated annotation clusters	Short-chain dehydrogenase/reductase	2.0		Cell adhesion	3.0	
				Follistatin-like proteins	2.5	
				Protein phosphatase binding	2.1	
				Embryonic development	1.5	
				Membrane organisation	1.4	

Down-regulated KEGG pathways/GO terms d	GO:0055114 oxidation-reduction		<0.0001	Cell adhesion molecules		0.001
	GO:0045833 regulation of lipid metabolic process		0.0047	Cardiomyopathy		0.005
	GO:0005932 microtubule basal body		0.0068			

a Enriched annotation terms among genes with altered regulation (based on genes with *p*_*f*_ < 0.01 and FC > 2 from the microarray analysis). In each annotation type the top five entries with score > 1 or *p* < 0.01 are shown.

b The significance of enrichment was measured by the *score*, the geometric mean (−log) of the *p*_*f*_-values of the contributing genes, or by the *p*-value.

c *p*-values were derived by a modified Fisher's exact test.

d No significant enrichment of genes in KEGG pathways was seen among down-regulated genes in the kidney, so the most significantly enriched gene ontology (GO) terms (*p* < 0.01) are given for this gene set.

**Table 4:** Pathway analysis (IPA, based on all probesets).

Classification	Kidney	Score^a^	*p*^a^	Molecules^b^	Lymph node	Score	*p*	Molecules
Top networks	Cellular assembly and organisation; genetic disorder; ophthalmic disease.	37			RNA post transcriptional modification; protein synthesis; small molecule biochemistry.	37		
	Antigen presentation; inflammatory response; genetic disorder.	35			Cell cycle; developmental disorder; genetic disorder.	37		
	DNA replication, recombination and repair; cellular assembly and organisation; cell–cell signalling and interaction.	35			Cancer; cell cycle; cellular growth and proliferation.	33		
	Amino acid metabolism; protein synthesis; small molecule biochemistry.	31			Cellular movement; nervous system development and function; cell morphology.	32		
	Cellular development; haematological system development and function; haematopoiesis.	31			Protein trafficking, RNA post-transcriptional modification, molecular transport.	32		

Top diseases/disorders	Inflammatory response.		0.0006	217	Cancer.		0.021	659
	Immunological disease.		0.0006	270	Genetic disorder.		0.020	247
	Inflammatory disease.		0.0006	292	Respiratory disease.		0.001	113
	connective tissue disorders.		0.0001	200	Gastrointestinal disease.		0.020	239
	skeletal and muscular disorders.		0.0002	275	Reproductive system disease.		0.020	367

Top molecular functions	Cellular growth and proliferation.		0.0005	254	Cell cycle.		0.021	291
	Cellular development.		0.0006	215	Cellular assembly and organisation.		0.014	79
	Cellular function and maintenance.		0.0005	136	DNA replication, recombination and repair.		0.019	164
	Cell death.		0.0004	292	Cellular movement.		0.020	191
	Cell–cell signalling and interaction.		0.0005	207	Antigen presentation.		0.014	33

Top canonical pathways	Antigen presentation.		<0.0001	16/43	Mitochondrial dysfunction.		<0.0001	42/169
	CD28 signalling in Th cells.		<0.0001	33/135	Protein ubiquitination pathway.		<0.0001	66/274
	iCOS–iCOSL signalling in T helper cells.		<0.0001	27/125	Ubiquinone biosynthesis.		<0.0001	26/119
	Natural Killer cell signalling.		<0.0001	25/116	Mitotic roles of polo-like kinase.		<0.0001	21/63
	Systemic Lupus Erythematosus signalling.		<0.0001	25/125	Cell cycle control of chromosomal replication.		<0.0001	14/31

a The *score* (integer; negative log of respective IPA *p*-value) or maximum *p*-value assigned by the IPA software for each classification is given. IPA uses a right tailed Fisher's exact test to calculate *p*-values.

b The number of molecules with significantly altered expression assigned to each classification from the submitted gene lists or the number of genes/total number of molecules in pathway.

## Appendix A Supplementary data

Supplementary Table S1 Known genes with at least 2-fold change between MCF-affected and control tissues in either kidney or lymph node. Fold-change in gene expression (FC) and *p*-values adjusted for false discovery rate (*p*_*f*_) are listed for kidney (K) and lymph node (LN) samples, in order of greatest fold-change in either tissue. Where FC and *p*_*f*_ data are not present, the corresponding gene expression changes were not found to be significant at the 99% level.
