# Supplementary material for: Host gene expression changes in cattle infected with Alcelaphine herpesvirus 1
Source: Virus Res. 2012 Oct;169(1):246–54. doi: 10.1016/j.virusres.2012.08.011 (PMC3657188; doi:10.1016/j.virusres.2012.08.011)
Supplement: Supplementary Table S1 — Known genes with at least 2-fold change between MCF-affected and control tissues in either kidney or lymph node. Fold-change in gene expression (FC) and p-values adjusted for false discovery rate (pf) are listed for kidney (K) and lymph node (LN) samples, in order of greatest fold-change in either tissue. Where FC and pf data are not present, the corresponding gene expression changes were not found to be significant at the 99% level. [file mmc1.doc]

**Supplementary Table S1**

Known genes with at least two- fold change between MCF-affected and control tissues in either kidney or lymph node. Fold-change in gene expression (FC) and p-values adjusted for false discovery rate (*p_f_*) are listed for kidney (K) and lymph node (LN) samples, in order of greatest fold-change in either tissue. Where FC and p*_f_* data are not present, the corresponding gene expression changes were not found to be significant at the 99% level.

| **Gene Symbol** | **Description** | **K (FC)** | **K (*p_f_*)** | **LN (FC)** | **LN (*p_f_*)** |
| --- | --- | --- | --- | --- | --- |
| CXCL10 | chemokine (C-X-C motif) ligand 10 | 14.6 | <0.0001 |  |  |
| GZMB | granzyme B |  |  | 10.0 | 0.0001 |
| AKR1C4 | aldo-keto reductase family 1, member C4 |  |  | 5.7 | 0.0016 |
| CCL8 | chemokine (C-C motif) ligand 8 | 5.0 | <0.0001 |  |  |
| TMEM156 | transmembrane protein 156 | 4.1 | <0.0001 |  |  |
| GBP4 | guanylate binding protein 4 | 4.0 | 0.0010 |  |  |
| BCL2A1 | BCL2-related protein A1 | 4.0 | <0.0001 |  |  |
| CCL19 | chemokine (C-C motif) ligand 19 | 4.0 | 0.0001 |  |  |
| CD3D | CD3d molecule, delta (CD3-TCR complex) (mean) | 3.8 | <0.0001 |  |  |
| SAMD9 | sterile alpha motif domain containing 9 | 3.7 | 0.0001 |  |  |
| CORO1A | coronin, actin binding protein, 1A | 3.7 | <0.0001 |  |  |
| BOLA | MHC class I heavy chain (mean) | 3.7 | 0.0088 |  |  |
| IGHM | immunoglobulin heavy constant mu | 3.7 | 0.0021 |  |  |
| PSMB9 | proteasome (prosome, macropain) subunit, beta type, 9 | 3.7 | <0.0001 |  |  |
| TAP1 | transporter 1, ATP-binding cassette, sub-family B (MDR/TAP) | 3.7 | <0.0001 |  |  |
| CD48 | CD48 molecule | 3.7 | <0.0001 |  |  |
| C3 | complement component 3 (mean) | 3.7 | 0.0051 |  |  |
| CLEC6A | C-type lectin domain family 6, member A | 3.7 | 0.0007 |  |  |
| KIAA0101 | KIAA0101 protein | 3.6 | 0.0002 |  |  |
| PTPRC | protein tyrosine phosphatase, receptor type, C | 3.6 | <0.0001 |  |  |
| LCP1 | lymphocyte cytosolic protein 1 (L-plastin) | 3.5 | <0.0001 |  |  |
| GIMAP7 | GTPase, IMAP family member 7 (mean) | 3.5 | 0.0021 |  |  |
| C1QB | complement component 1, q subcomponent, B chain | 3.5 | <0.0001 |  |  |
| CD53 | CD53 molecule | 3.5 | <0.0001 |  |  |
| CD3G | CD3g molecule, gamma (CD3-TCR complex) | 3.5 | <0.0001 |  |  |
| CD180 | CD180 molecule | 3.4 | <0.0001 |  |  |
| CXCR6 | chemokine (C-X-C motif) receptor 6 | 3.3 | <0.0001 |  |  |
| ITGB2 | integrin, beta 2 (complement component 3 receptor 3 and 4 subunit) | 3.3 | <0.0001 |  |  |
| PTPN22 | protein tyrosine phosphatase, non-receptor type 22 (lymphoid) | 3.3 | 0.0001 |  |  |
| HSPH1 | heat shock 105kDa/110kDa protein 1 |  |  | 3.2 | 0.0004 |
| C1QC | complement component 1, q subcomponent, C chain | 3.1 | <0.0001 |  |  |
| NKG7 | natural killer cell group 7 sequence | 3.1 | <0.0001 |  |  |
| C1S | complement component 1, s subcomponent | 3.1 | 0.0001 |  |  |
| IKZF3 | IKAROS family zinc finger 3 (Aiolos) | 3.0 | <0.0001 |  |  |
| HSPCA | heat shock 90kD protein 1, alpha |  |  | 3.0 | 0.0009 |
| TRB@ | T cell receptor, beta cluster | 3.0 | <0.0001 |  |  |
| TMSB10 | thymosin beta 10 | 3.0 | <0.0001 |  |  |
| LCP2 | lymphocyte cytosolic protein 2 (SH2 domain containing leukocyte protein of 76kDa) | 3.0 | 0.0001 |  |  |
| NCF1 | neutrophil cytosolic factor 1 | 2.9 | <0.0001 |  |  |
| BOLA-DMB | major histocompatibility complex, class II, DM beta-chain, expressed | 2.9 | <0.0001 |  |  |
| TEX12 | testis expressed 12 | 2.9 | 0.0007 |  |  |
| RAC2 | ras-related C3 botulinum toxin substrate 2 (rho family, small GTP binding protein Rac2) | 2.9 | <0.0001 |  |  |
| CLEC2D | C-type lectin domain family 2, member D | 2.9 | <0.0001 |  |  |
| CD40 | CD40 molecule, TNF receptor superfamily member 5 | 2.9 | <0.0001 |  |  |
| LAPTM5 | lysosomal protein transmembrane 5 | 2.8 | <0.0001 |  |  |
| PEX5 | peroxisomal biogenesis factor 5 | 2.8 | 0.0099 |  |  |
| BoLA-DRB3 | major histocompatibility complex, class II, DRB3 | 2.8 | <0.0001 |  |  |
| DMBT1 | deleted in malignant brain tumors 1 |  |  | 2.8 | 0.0008 |
| LCK | lymphocyte-specific protein tyrosine kinase (mean) | 2.8 | <0.0001 |  |  |
| TIMD4 | T-cell immunoglobulin and mucin domain containing 4 | 2.8 | 0.0003 |  |  |
| PSMB10 | proteasome (prosome, macropain) subunit, beta type, 10 | 2.8 | 0.0001 |  |  |
| ZAP70 | zeta-chain (TCR) associated protein kinase 70kDa | 2.8 | <0.0001 |  |  |
| C1QA | complement component 1, q subcomponent, A chain | 2.8 | <0.0001 |  |  |
| RNASE6 | ribonuclease, RNase A family, k6 | 2.8 | 0.0002 |  |  |
| PLEK | pleckstrin | 2.7 | <0.0001 |  |  |
| IRF1 | interferon regulatory factor 1 | 2.7 | <0.0001 |  |  |
| MSR1 | macrophage scavenger receptor 1 (mean) | 2.7 | 0.0022 |  |  |
| CCNB1 | cyclin B1 |  |  | 2.7 | 0.0004 |
| PSPH | phosphoserine phosphatase (mean) |  |  | 2.7 | 0.0025 |
| LAT | linker for activation of T cells | 2.7 | <0.0001 |  |  |
| HCLS1 | hematopoietic cell-specific Lyn substrate 1 (mean) | 2.7 | 0.0003 |  |  |
| SLAMF7 | SLAM family member 7 | 2.7 | <0.0001 |  |  |
| CTSS | cathepsin S | 2.7 | <0.0001 |  |  |
| PRKCB | protein kinase C, beta (mean) | 2.7 | <0.0001 |  |  |
| ARHGDIB | Rho GDP dissociation inhibitor (GDI) beta | 2.7 | <0.0001 |  |  |
| TAP2 | transporter 2, ATP-binding cassette, sub-family B (MDR/TAP) | 2.7 | 0.0002 |  |  |
| CD8B | CD8b molecule | 2.7 | <0.0001 |  |  |
| CHI3L1 | chitinase 3-like 1 (cartilage glycoprotein-39) | 2.7 | 0.0062 |  |  |
| ANKRD22 | ankyrin repeat domain 22 (mean) |  |  | 2.6 | 0.0026 |
| LRMP | lymphoid-restricted membrane protein | 2.6 | <0.0001 |  |  |
| PSMB8 | proteasome (prosome, macropain) subunit, beta type, 8 (large multifunctional peptidase 7) | 2.6 | <0.0001 |  |  |
| TMEM37 | transmembrane protein 37 | 2.6 | 0.0002 |  |  |
| CYBB | cytochrome b-245, beta polypeptide (mean) | 2.6 | 0.0003 |  |  |
| ASPM | asp (abnormal spindle) homolog, microcephaly associated (Drosophila) | 2.5 | 0.0057 |  |  |
| CD69 | CD69 molecule | 2.5 | 0.0002 |  |  |
| MPEG1 | macrophage expressed 1 | 2.5 | 0.0001 |  |  |
| BOLA-DMA | major histocompatibility complex, class II, DM alpha-chain, expressed | 2.5 | <0.0001 |  |  |
| MT2A | metallothionein 2A |  |  | 2.5 | 0.0065 |
| BOLA-DRA | major histocompatibility complex, class II, DR alpha | 2.5 | <0.0001 |  |  |
| TNFSF13B | tumor necrosis factor (ligand) superfamily, member 13b (mean) | 2.5 | 0.0004 |  |  |
| ERCC6L | excision repair cross-complementing rodent repair deficiency, complementation group 6-like |  |  | 2.5 | 0.0007 |
| SLAMF9 | SLAM family member 9 | 2.5 | 0.0013 |  |  |
| CENPF | centromere protein F, 350/400ka (mitosin) | 2.5 | 0.0002 |  |  |
| CD74 | CD74 molecule, major histocompatibility complex, class II invariant chain | 2.5 | 0.0010 |  |  |
| APBB1IP | amyloid beta (A4) precursor protein-binding, family B, member 1 interacting protein | 2.5 | <0.0001 |  |  |
| LAP3 | leucine aminopeptidase 3 |  |  | 2.5 | 0.0002 |
| CD68 | CD68 molecule | 2.4 | 0.0001 |  |  |
| CD2 | CD2 molecule (mean) | 2.4 | 0.0003 |  |  |
| P2RY10 | purinergic receptor P2Y, G-protein coupled, 10 | 2.4 | <0.0001 |  |  |
| AIF1 | allograft inflammatory factor 1 | 2.4 | <0.0001 |  |  |
| CASP4 | caspase 4, apoptosis-related cysteine peptidase | 2.4 | <0.0001 |  |  |
| ITGB7 | integrin, beta 7 | 2.4 | <0.0001 |  |  |
| ORC1L | origin recognition complex, subunit 1-like (yeast) |  |  | 2.4 | 0.0004 |
| PARP14 | poly (ADP-ribose) polymerase family, member 14 | 2.3 | 0.0026 |  |  |
| FCGR2B | Fc fragment of IgG, low affinity IIb, receptor (CD32) | 2.3 | 0.0007 |  |  |
| GBP5 | guanylate binding protein 5 (mean) | 2.3 | 0.0073 |  |  |
| BIN2 | bridging integrator 2 | 2.3 | <0.0001 |  |  |
| CTSW | cathepsin W | 2.3 | 0.0002 |  |  |
| CDCA2 | cell division cycle associated 2 |  |  | 2.3 | 0.0024 |
| SOD2 | superoxide dismutase 2, mitochondrial |  |  | 2.3 | 0.0095 |
| FERMT3 | fermitin family homolog 3 (Drosophila) | 2.3 | <0.0001 |  |  |
| FBXO5 | F-box protein 5 |  |  | 2.3 | 0.0005 |
| LPXN | leupaxin | 2.3 | <0.0001 |  |  |
| ARHGAP30 | Rho GTPase activating protein 30 | 2.3 | <0.0001 |  |  |
| SLC16A6 | solute carrier family 16, member 6 (monocarboxylic acid transporter 7) |  |  | 2.3 | 0.0013 |
| SLAMF8 | SLAM family member 8 |  |  | 2.3 | 0.0035 |
| LAG3 | lymphocyte-activation gene 3 |  |  | 2.3 | 0.0008 |
| SMC2 | structural maintenance of chromosomes 2 |  |  | 2.3 | 0.0025 |
| NAT13 | N-acetyltransferase 13 (GCN5-related) |  |  | 2.2 | 0.0004 |
| RGS10 | regulator of G-protein signaling 10 | 2.2 | 0.0001 |  |  |
| SELL | selectin L | 2.2 | <0.0001 |  |  |
| CD14 | CD14 molecule | 2.2 | 0.0056 |  |  |
| HCK | hemopoietic cell kinase | 2.2 | 0.0001 |  |  |
| CTSH | cathepsin H | 2.2 | <0.0001 |  |  |
| LIPG | lipase, endothelial |  |  | 2.2 | 0.0021 |
| UBE2S | ubiquitin-conjugating enzyme E2S |  |  | 2.2 | 0.0003 |
| MYO1F | myosin IF | 2.2 | <0.0001 |  |  |
| CD83 | CD83 molecule | 2.2 | 0.0004 |  |  |
| POLE2 | polymerase (DNA directed), epsilon 2 (p59 subunit) |  |  | 2.2 | 0.0009 |
| B2M | beta-2-microglobulin | 2.2 | <0.0001 |  |  |
| ADCY7 | adenylate cyclase 7 | 2.2 | <0.0001 |  |  |
| CD84 | CD84 molecule | 2.2 | 0.0043 |  |  |
| PDIA4 | protein disulfide isomerase family A, member 4 |  |  | 2.2 | 0.0010 |
| BUB1 | budding uninhibited by benzimidazoles 1 homolog (yeast) |  |  | 2.2 | 0.0021 |
| MST4 | serine/threonine protein kinase MST4 | 2.2 | 0.0010 |  |  |
| AURKA | aurora kinase A (mean) |  |  | 2.2 | 0.0017 |
| TCRG | T-cell receptor gamma chain (mean) |  |  | 2.2 | 0.0012 |
| IFI16 | interferon, gamma-inducible protein 16 | 2.1 | 0.0019 |  |  |
| SAMSN1 | SAM domain, SH3 domain and nuclear localization signals 1 | 2.1 | 0.0017 |  |  |
| LY9 | lymphocyte antigen 9 | 2.1 | 0.0003 |  |  |
| TPX2 | TPX2, microtubule-associated, homolog (Xenopus laevis) |  |  | 2.1 | 0.0009 |
| MRM1 | mitochondrial rRNA methyltransferase 1 homolog (S. cerevisiae) |  |  | 2.1 | 0.0032 |
| FCGR1A | Fc fragment of IgG, high affinity Ia, receptor (CD64) | 2.1 | 0.0003 |  |  |
| TIMP2 | TIMP metallopeptidase inhibitor 2 | 2.1 | 0.0022 |  |  |
| XDH | xanthine dehydrogenase (mean) | 2.1 | 0.0019 |  |  |
| FKBP4 | FK506 binding protein 4, 59kDa |  |  | 2.1 | 0.0004 |
| TIFA | TRAF-interacting protein with forkhead-associated domain | 2.1 | 0.0002 |  |  |
| DOCK10 | dedicator of cytokinesis 10 | 2.1 | 0.0001 |  |  |
| CYB5B | cytochrome b5 type B (outer mitochondrial membrane) |  |  | 2.1 | 0.0006 |
| CD44 | CD44 molecule (Indian blood group) | 2.1 | 0.0001 |  |  |
| GMFG | glia maturation factor, gamma | 2.1 | 0.0001 |  |  |
| BAZ1A | bromodomain adjacent to zinc finger domain, 1A | 2.1 | 0.0001 |  |  |
| SEMA4A | sema domain, immunoglobulin domain (Ig), transmembrane domain (TM) and short cytoplasmic domain, (semaphorin) 4A | 2.1 | 0.0001 |  |  |
| MELK | maternal embryonic leucine zipper kinase | 2.1 | 0.0022 |  |  |
| PLXNC1 | plexin C1 | 2.1 | 0.0035 |  |  |
| MKI67 | antigen identified by monoclonal antibody Ki-67 |  |  | 2.1 | 0.0008 |
| IL18 | interleukin 18 (interferon-gamma-inducing factor) | 2.1 | 0.0002 |  |  |
| CD86 | CD86 molecule | 2.1 | 0.0001 |  |  |
| TOX2 | TOX high mobility group box family member 2 | 2.1 | 0.0012 |  |  |
| ITGAL | integrin, alpha L (antigen CD11A (p180), lymphocyte function-associated antigen 1; alpha polypeptide) | 2.1 | 0.0004 |  |  |
| SKAP1 | src kinase associated phosphoprotein 1 (mean) | 2.1 | 0.0001 |  |  |
| ESPL1 | extra spindle pole bodies homolog 1 (S. cerevisiae) |  |  | 2.1 | 0.0009 |
| SRPX | sushi-repeat-containing protein, X-linked | 2.1 | 0.0029 |  |  |
| SLC11A1 | solute carrier family 11 (proton-coupled divalent metal ion transporters), member 1 |  |  | 2.1 | 0.0032 |
| PDXK | pyridoxal (pyridoxine, vitamin B6) kinase |  |  | 2.1 | 0.0007 |
| NCAPH | non-SMC condensin I complex, subunit H | 2.1 | 0.0023 |  |  |
| SLC25A5 | solute carrier family 25 (mitochondrial carrier; adenine nucleotide translocator), member 5 |  |  | 2.0 | 0.0005 |
| CST7 | cystatin F (leukocystatin) | 2.0 | <0.0001 |  |  |
| SH3KBP1 | SH3-domain kinase binding protein 1 | 2.0 | <0.0001 |  |  |
| BIRC3 | baculoviral IAP repeat-containing 3 | 2.0 | 0.0025 |  |  |
| VPS36 | vacuolar protein sorting 36 homolog (S. cerevisiae) |  |  | 2.0 | 0.0003 |
| TRAF3IP3 | TRAF3 interacting protein 3 | 2.0 | 0.0001 |  |  |
| WDFY4 | WDFY family member 4 | 2.0 | 0.0022 |  |  |
| TGM2 | transglutaminase 2 (C polypeptide, protein-glutamine-gamma-glutamyltransferase) | 2.0 | 0.0008 |  |  |
| NMI | N-myc (and STAT) interactor | 2.0 | 0.0004 |  |  |
| INPP5D | inositol polyphosphate-5-phosphatase, 145kDa | 2.0 | <0.0001 |  |  |
| FLRT2 | similar to KIAA0405 |  |  | 0.5 | 0.0009 |
| ELL3 | elongation factor RNA polymerase II-like 3 |  |  | 0.5 | 0.0046 |
| MSRB2 | methionine sulfoxide reductase B2 | 0.5 | 0.0002 |  |  |
| SPARCL1 | SPARC-like 1 (hevin) |  |  | 0.5 | 0.0046 |
| CAPRIN2 | caprin family member 2 |  |  | 0.5 | 0.0005 |
| FAH | fumarylacetoacetate hydrolase (fumarylacetoacetase) | 0.5 | 0.0001 |  |  |
| CAPG | capping protein (actin filament), gelsolin-like |  |  | 0.5 | 0.0041 |
| NAV3 | neuron navigator 3 |  |  | 0.5 | 0.0010 |
| NCEH1 | neutral cholesterol ester hydrolase 1 |  |  | 0.5 | 0.0011 |
| TTC36 | tetratricopeptide repeat domain 36 | 0.5 | 0.0002 |  |  |
| KLF4 | Kruppel-like factor 4 (gut) |  |  | 0.5 | 0.0041 |
| ABCC3 | ATP-binding cassette, sub-family C (CFTR/MRP), member 3 |  |  | 0.5 | 0.0058 |
| DTNB | dystrobrevin, beta | 0.5 | 0.0003 |  |  |
| RORC | RAR-related orphan receptor C | 0.5 | 0.0019 |  |  |
| MME | membrane metallo-endopeptidase |  |  | 0.5 | 0.0041 |
| DAB2 | disabled homolog 2, mitogen-responsive phosphoprotein (Drosophila) |  |  | 0.5 | 0.0069 |
| CLGN | calmegin (mean) |  |  | 0.5 | 0.0016 |
| CDH2 | cadherin 2, type 1, N-cadherin (neuronal) |  |  | 0.5 | 0.0022 |
| MXRA7 | matrix-remodelling associated 7 |  |  | 0.5 | 0.0005 |
| PLBD1 | phospholipase B domain containing 1 |  |  | 0.5 | 0.0020 |
| ECRG4 | esophageal cancer related gene 4 protein |  |  | 0.5 | 0.0021 |
| DDO | D-aspartate oxidase | 0.5 | 0.0011 |  |  |
| ASPDH | aspartate dehydrogenase domain containing | 0.5 | 0.0024 |  |  |
| GPM6A | glycoprotein M6A |  |  | 0.5 | 0.0037 |
| HOXA9 | homeobox A9 |  |  | 0.5 | 0.0012 |
| ADM | adrenomedullin |  |  | 0.5 | 0.0083 |
| SDC2 | syndecan 2 |  |  | 0.5 | 0.0061 |
| TLE1 | transducin-like enhancer of split 1 (E(sp1) homolog, Drosophila) |  |  | 0.5 | 0.0003 |
| PKIG | protein kinase (cAMP-dependent, catalytic) inhibitor gamma |  |  | 0.5 | 0.0005 |
| SCN4B | sodium channel, voltage-gated, type IV, beta |  |  | 0.5 | 0.0002 |
| ZMYND12 | zinc finger, MYND-type containing 12 | 0.5 | 0.0002 |  |  |
| CH25H | cholesterol 25-hydroxylase |  |  | 0.5 | 0.0091 |
| IRX3 | iroquois homeobox 3 |  |  | 0.5 | 0.0014 |
| ERBB3 | v-erb-b2 erythroblastic leukemia viral oncogene homolog 3 (avian) | 0.5 | 0.0011 |  |  |
| MS4A1 | membrane-spanning 4-domains, subfamily A, member 1 (mean) |  |  | 0.5 | 0.0068 |
| H19 | H19, imprinted maternally expressed transcript (non-protein coding) | 0.4 | 0.0074 |  |  |
| HVCN1 | hydrogen voltage-gated channel 1 |  |  | 0.4 | 0.0014 |
| DSC2 | desmocollin 2 |  |  | 0.4 | 0.0004 |
| CXXC5 | CXXC finger 5 |  |  | 0.4 | 0.0011 |
| LY6D | lymphocyte antigen 6 complex, locus D |  |  | 0.4 | 0.0084 |
| APOM | apolipoprotein M (mean) | 0.4 | 0.0001 |  |  |
| DUSP1 | dual specificity phosphatase 1 |  |  | 0.4 | 0.0038 |
| CCL21 | chemokine (C-C motif) ligand 21 |  |  | 0.4 | 0.0008 |
| COL12A1 | collagen, type XII, alpha 1 |  |  | 0.4 | 0.0005 |
| NAPSA | napsin A aspartic peptidase |  |  | 0.4 | 0.0010 |
| CCRL1 | chemokine (C-C motif) receptor-like 1 |  |  | 0.4 | 0.0003 |
| SLC27A2 | solute carrier family 27 (fatty acid transporter), member 2 | 0.4 | 0.0019 |  |  |
| hare | hyaluronan receptor for endocytosis-like |  |  | 0.4 | 0.0027 |
| APOH | apolipoprotein H (beta-2-glycoprotein I) | 0.4 | 0.0013 |  |  |
| MEST | mesoderm specific transcript homolog (mouse) |  |  | 0.4 | 0.0005 |
| MEOX2 | mesenchyme homeobox 2 |  |  | 0.4 | 0.0011 |
| APOC2 | apolipoprotein C-II | 0.4 | 0.0023 |  |  |
| SCD5 | stearoyl-CoA desaturase 5 |  |  | 0.4 | 0.0004 |
| CCL20 | chemokine (C-C motif) ligand 20 |  |  | 0.4 | 0.0004 |
| PROS1 | protein S (alpha) |  |  | 0.4 | 0.0020 |
| DHRS12 | dehydrogenase/reductase (SDR family) member 12 | 0.4 | <0.0001 |  |  |
| CLEC4G | C-type lectin domain family 4, member G |  |  | 0.4 | 0.0023 |
| DAPL1 | death associated protein-like 1 |  |  | 0.3 | 0.0001 |
| F13A1 | coagulation factor XIII, A1 polypeptide (mean) |  |  | 0.3 | 0.0060 |
| SHISA3 | shisa homolog 3 (Xenopus laevis) |  |  | 0.3 | 0.0009 |
| AGXT2L1 | alanine-glyoxylate aminotransferase 2-like 1 | 0.3 | 0.0001 |  |  |
| SELE | selectin E |  |  | 0.3 | 0.0007 |
| ACE2 | angiotensin I converting enzyme (peptidyl-dipeptidase A) 2 | 0.3 | <0.0001 |  |  |
| PCK2 | phosphoenolpyruvate carboxykinase 2 (mitochondrial) | 0.3 | 0.0001 |  |  |
| RNASE1 | ribonuclease |  |  | 0.3 | 0.0004 |
| ALOX5AP | arachidonate 5-lipoxygenase-activating protein |  |  | 0.3 | 0.0003 |
| SPP1 | secreted phosphoprotein 1 |  |  | 0.3 | 0.0008 |
| GSTA1 | glutathione S-transferase alpha 1 | 0.2 | <0.0001 |  |  |
